# Acute retinal necrosis following recombinant subunit varicella-zoster virus vaccine

**DOI:** 10.1016/j.ajoc.2020.100962

**Published:** 2020-10-08

**Authors:** Rebecca I. Chen, Jordan D. Deaner, Sunil K. Srivastava, Careen Y. Lowder

**Affiliations:** Cleveland Clinic Cole Eye Institute, 9500 Euclid Avenue i-32 Cleveland, OH, 44195, USA

**Keywords:** Acute retinal necrosis, Varicella-zoster virus, Zoster vaccine, Recombinant zoster subunit vaccine, Shingrix

## Abstract

**Purpose:**

Previously, secondary prevention of herpes zoster required live-attenuated vaccination, which is contraindicated in immunocompromised populations. More recently, a recombinant subunit vaccine (Shingrix, GlaxoSmithKline, Research Triangle Park, North Carolina) was approved by the Food and Drug Administration. Iatrogenic varicella-zoster virus (VZV) infection is theoretically impossible as it does not contain a live virus. We present a case of acute retinal necrosis (ARN) and disseminated zoster after receiving the recombinant subunit vaccine.

**Observations:**

A 65-year-old woman with past medical history of multiple myeloma treated with a previous autologous hematopoietic stem cell transplant and now with daratumumab and pomalidomide developed disseminated zoster and subsequently acute retinal necrosis weeks after receiving the zoster subunit vaccine. Molecular testing confirmed the presence of VZV, and the absence of herpes simplex virus, cytomegalovirus, and toxoplasmosis. The VZV was found to be genotypically wildtype and not related to the Oka strain used in the live-attenuated zoster vaccine. She was treated with systemic valacyclovir and intravitreal foscarnet.

**Conclusions and importance:**

This is the first report of VZV infection following the zoster subunit vaccine. The Advisory Committee on Immunization Practices (ACIP) has recommended the recombinant subunit vaccine over the live-attenuated vaccine due to its superior efficacy. The off-label use of the subunit vaccine in immunocompromised populations has been supported up to this point by studies demonstrating its relative safety. Though post-vaccination VZV infection or reactivation appears to be rare, clinicians should be aware of this potential complication to the recombinant subunit vaccine.

## 1 Introduction

Herpes zoster is the result of reactivation of the varicella-zoster virus (VZV) from its latent state in nerve ganglion.^1^^,^^2^ Reactivation of VZV is associated with a decline in cell mediated immunity that occurs with natural aging or an acquired immunocompromised state.^3^, ^4^, ^5^ Herpes zoster most commonly presents as a painful localized rash confined to a single dermatome.^6^ However, a myriad of neurologic, ophthalmic, and severe systemic manifestations have been reported and can be associated with significant mortality and morbidity.^3^^,^^6^^,^^7^ Previously, immunization was only possible with live-attenuated VZV vaccination.^4^^,^^8^ In 2017, the Food and Drug Administration (FDA) approved a recombinant subunit vaccine for herpes zoster (Shingrix, GlaxoSmithKline, Research Triangle Park, North Carolina).^4^^,^^8^ We present a case of acute retinal necrosis (ARN) and disseminated varicella zoster infection after receiving the recombinant subunit vaccine.

### 1.1 Case report

A 65-year-old woman presented with 2-week history of worsening floaters and blurred vision in her left eye (OS) for 5 days. She had a past medical history of multiple myeloma treated with a previous autologous hematopoietic stem cell transplant and now with daratumumab and pomalidomide. Notably, she received the first dose of the recombinant zoster subunit vaccine (Shingrix) 2 months prior to presentation. Six weeks after receiving the vaccine, she was hospitalized after developing a systemic vesicular rash and hypoxic respiratory failure, during which she was treated for disseminated varicella zoster and viral pneumonia. She was treated with intravenous acyclovir 10 mg/kg every 8 hours and discharged on oral acyclovir 400 mg twice daily.

**Fig. 1:**
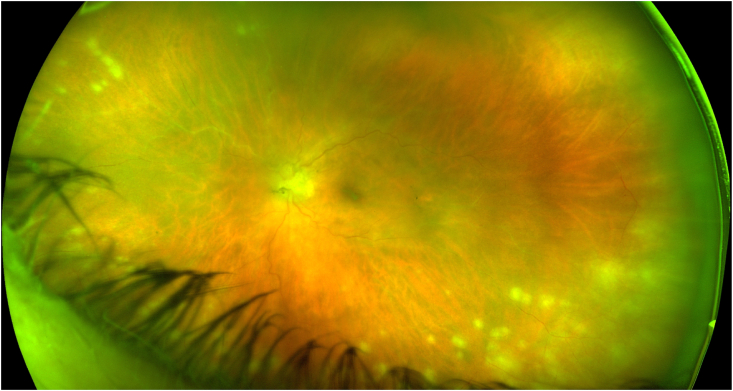
Fundus photograph of the patient's left eye demonstrated a hazy media with inferotemporal and superonasal multifocal areas of retinal whitening with associated artery sheathing.

Two weeks after discharge from the hospital, she developed floaters, followed by progressive blurry vision (OS). On exam, her best corrected visual acuity was 20/25 in the unaffected right eye (OD) and 20/70–1 in the left eye. She had a left afferent pupillary defect and intraocular pressures of 14 and 21 mmHg in her right and left eyes, respectively. Full ocular examination of the right eye was unremarkable. Slit lamp examination of the left eye revealed 2+ anterior chamber cells and 2+ anterior vitreous cells. Funduscopic exam OS demonstrated 2+ vitreous haze and peripheral multifocal areas of retinal whitening with associated artery sheathing (Fig. 1).

An anterior chamber paracentesis was performed on the left eye due to the high suspicion of viral retinitis based on her presentation. Her aqueous was sent for polymerase chain reaction (PCR) detection of VZV, herpes simplex virus (HSV), cytomegalovirus (CMV), and toxoplasmosis. The patient was given an intravitreal injection of foscarnet (2.4 mg/0.1 ml) and started on valacyclovir 2 g three times per day. The patient was asked to stop her immunotherapy. Five days after presentation, PCR testing for HSV, CMV, and toxoplasmosis DNA resulted as undetectable. VZV DNA was detected. Genotyping performed by the Centers for Disease Control (CDC) identified the virus as wild-type and not associated with the Oka strain used to manufacture the live attenuated vaccine.

**Fig. 2:**
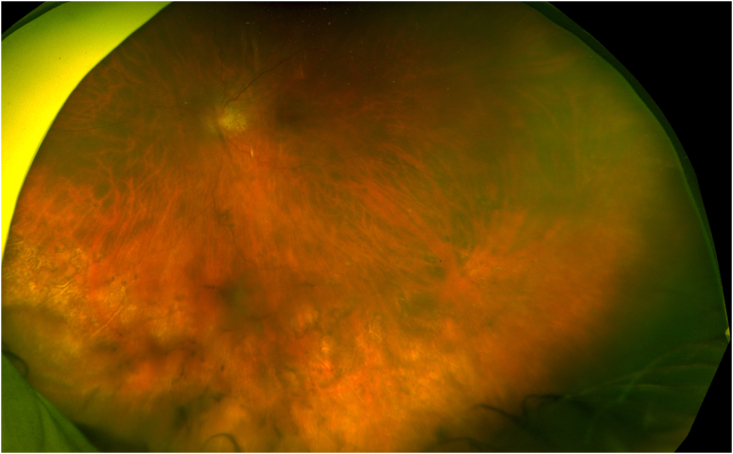
Fundus photograph of the patient's left eye 19 weeks after initial presentation showing resolution of retinitis with large areas of peripheral atrophy.

The patient was continued on therapeutic dose valacyclovir and treated with biweekly intravitreal injections of foscarnet for a total of 11 weeks. At week 8, there was consistent regression of retinitis, and the patient was asked to restart her immunotherapy for multiple myeloma. At her most recent follow-up, 19 weeks after presentation, her examination remains free of active retinitis (Fig. 2). Her visual acuity had improved to 20/50+ with resolution of her afferent pupillary defect.

## 2 Discussion

There are several FDA-approved vaccinations for the prevention of VZV. In children, Varivax (Merck, Whitehouse Station, New Jersey) is a live attenuated virus used for primary prevention of varicella.^9^ For the secondary prevention of zoster, Zostavax (Merck, Whitehouse Station, New Jersey) is a live attenuated Oka strain virus that was FDA approved in 2006, with nearly 22 million doses dispensed in the United States (as of 2015).^8^^,^^9^ However, its use has been limited due to waning immunogenicity over time and risk of zoster reactivation in immunocompromised individuals.^8^ Shingrix, a recombinant subunit zoster vaccine, was FDA approved in 2017 for zoster prevention in immunocompetent adults aged 50 years and older and has subsequently been administered in 11 million patients (as of September 2019).^4^^,^^8^^,^^11^ It contains the VZV glycoprotein E (gE) subunit antigen, which is the most abundant cell surface marker of infected cells and is required for VZV replication.^4^ This antigenic subunit is produced using genetically engineered Chinese hamster ovary cells and subsequently purified.^12^ Importantly, varicella-zoster virus is not used in the manufacturing process and therefore eliminates the risk of iatrogenically induced zoster infection. The vaccine also includes the adjuvant AS01_B_, which is formulated with liposomes and 2 immunostimulants. 3-*O*-desacyl-4′-monophosphoryl lipid A activates innate immunity and cytokine production, while QS-21 stimulates antigen-specific antibody responses and T cell-mediated immunity.^4^^,^^8^^,^^13^ These immunostimulants synergistically increase gE-specific CD4^+^ T cell responses to vaccination.^13^ It is given as a 2-dose vaccine series, with the second dose given 2–6 months later to aid in long-term immunity, though increased humoral and cell-mediated immunogenicity markers have been observed after the first dose.^4^^,^^14^ Although the vaccine does not have FDA approval for this indication, Phase III trials have demonstrated safety and efficacy of the vaccine in various immunocompromised populations including those with multiple myeloma and hematopoietic stem cell transplant recipients.^14^^,^^15^

Ocular adverse events following any VZV vaccination are rare. There is a single case report of a 16-year-old girl developing anterior uveitis associated and a vesicular rash after receiving Varivax.^16^ There have been few reports of Zostavax causing ophthalmic adverse events including reactivation of VZV associated uveitis; ocular infection by reactivated, dormant wild-type virus; and primary ocular infection by vaccine strain virus. Two reported cases of interstitial keratitis following Zostavax administration suggest that the live attenuated vaccine can cause an unintended reactivation of a previously controlled immune response.^17^^,^^18^ Only 6 cases of presumed Zostavax associated viral retinitis have been reported in the literature.^19^, ^20^, ^21^, ^22^, ^23^ Charkoudian et al. were the first to report 2 cases of ARN following Zostavax administration in a 76-year-old woman with end stage renal disease and an 80-year-old man with renal transplant on immunosuppression, 6 days and 2 months after receiving the vaccine, respectively.^19^ Both cases were confirmed infections with VZV by PCR, but genotyping was not performed. Gonzales et al. were the first to report a case of ARN caused by genotype-confirmed Oka vaccine strain virus in a 20-year-old immunocompromised man.^20^ More recently, Menghini et al. reported a case of ARN after Zostavax in a 76-year-old man with a history of chronic lymphocytic leukemia that was confirmed to be wild-type VZV by genotyping.^22^

Our patient developed a systemic vesicular rash and viral pneumonia 6 weeks after Shingrix, followed by ARN secondary to VZV. As Shingrix is a subunit vaccine and not an attenuated live virus, iatrogenic infection is theoretically impossible. In our patient's case, the VZV isolate was genotypically confirmed to be wild-type, without evidence of recombination or mutation. The temporal relationship of this patient's disseminated zoster and ARN shortly following receipt of the recombinant subunit vaccine raises the possibility of an immunomodulatory phenomenon contributing to dormant VZV reactivation. It is also possible that the disseminated zoster and ARN are not related to the Shingrix vaccine.

Varicella activation or reactivation after any form of VZV vaccination is rare. In the Shingles Prevention Study, only 17 of the 19,270 patients who received the live attenuated Oka vaccine reported a varicella-like rash, with only 5 testing positive for wild-type VZV by PCR and none for Oka strain.^24^ In comparison, the efficacy study that prompted FDA approval of the VZV recombinant subunit vaccine reported a high rate of injection-site reaction 81.5% with pain and myalgia, but no patient developed a varicella-like rash.^25^ Post-licensure safety monitoring through the Vaccine Adverse Event Reporting System (VAERS) reported rates of post-immunization herpes zoster of 6.1 cases per 100,000 (196/3,200,000) patients following Shingrix (October 2017–June 2018) compared to 12.7 cases per 100,000 (2781/21,846,030) patients following Zostavax (May 2006–February 2015).^10^^,^^26^

Among those who had received Shingrix, VAERS also reported a rate of post-immunization inflammatory eye disease at 0.4 per 100,000 patients.^26^ Of the 13 patients reported with post-vaccination inflammatory eye disease, 9 developed herpes zoster near the eye with subsequent ocular involvement, 2 developed primary herpes zoster iridocyclitis, and 1 report each of ocular herpes zoster and herpes zoster keratoconjunctivitis.^26^ Our patient is the first reported case of disseminated zoster and ARN occurring shortly after Shingrix administration amongst 11 million patients who received at least one dose in the United States.^11^

## 3 Conclusions

In conclusion, our case of disseminated zoster and acute retinal necrosis following vaccination with the recombinant subunit has important clinical implications. The Advisory Committee on Immunization Practices (ACIP) has recommended the use of the recombinant gE vaccine over the live-attenuated Zostavax vaccine for secondary prevention of herpes zoster due to its wider range of efficacy.^4^^,^^27^ Additionally, there is significant interest in the recombinant vaccine's potential to benefit immunosuppressed patients in whom Zostavax is contraindicated, and the off-label use of Shingrix in these populations has been supported up to this point by studies demonstrating its relative safety in hematopoietic stem cell or solid organ transplant recipients.^12^^,^^13^^,^^28^ Though post-vaccination VZV infection or reactivation appears to be rare, clinicians should be aware of this potential complication to the recombinant subunit vaccine.

### 3.1 Patient consent

Consent to publish the case report was not obtained. This report does not contain any personal information that could lead to the identification of the patient.

## Funding

Unrestricted Grant Award from Research to Prevent Blindness to the Department of Ophthalmology at Cole Eye Institute (RPB1508DM).

## The nature of potential conflict of interest is described below

No conflict of interest exists.

We wish to confirm that there are no known conflicts of interest associated with this publication and there has been no significant financial support for this work that could have influenced its outcome.

Study investigator Sunil Srivastava reports the following financial relationships which are not relevant to this case study manuscript and pose no conflicts of interest.

## Funding

Funding was received for this work.

All of the sources of funding for the work described in this publication are acknowledged below:

Unrestricted Grant Award from Research to Prevent Blindness to the Department of Ophthalmology at Cole Eye Institute (RPB1508DM). Sunil Srivastava: research grants from Bausch and Lomb, Allergan, Novartis, Clearside, Zeiss, Sanofi, SantenGrant Funding: Ohio Department of Development TECH-13-059.

As this manuscript is a case report, the funding received by the institution and/or study investigators did not affect any study design, analysis, or preparation of this manuscript. No funding was received for this work.

### Intellectual property

We confirm that we have given due consideration to the protection of intellectual property associated with this work and that there are no impediments to publication, including the timing of publication, with respect to intellectual property. In so doing we confirm that we have followed the regulations of our institutions concerning intellectual property.

### Research ethics

We further confirm that any aspect of the work covered in this manuscript that has involved human patients has been conducted with the ethical approval of all relevant bodies and that such approvals are acknowledged within the manuscript.

IRB approval was obtained (required for studies and series of 3 or more cases)

Written consent to publish potentially identifying information, such as details or the case and photographs, was obtained from the patient(s) or their legal guardian(s).

## Authorship

The International Committee of Medical Journal Editors (ICMJE) recommends that authorship be based on the following four criteria:

1.Substantial contributions to the conception or design of the work; or the acquisition, analysis, or interpretation of data for the work; AND
2.Drafting the work or revising it critically for important intellectual content; AND
3.Final approval of the version to be published; AND
4.Agreement to be accountable for all aspects of the work in ensuring that questions related to the accuracy or integrity of any part of the work are appropriately investigated and resolved.

All those designated as authors should meet all four criteria for authorship, and all who meet the four criteria should be identified as authors. For more information on authorship, please see http://www.icmje.org/recommendations/browse/roles-and-responsibilities/defining-the-role-of-authors-and-contributors.html#two.

All listed authors meet the ICMJE criteria.  We attest that all authors contributed significantly to the creation of this manuscript, each having fulfilled criteria as established by the ICMJE.

One or more listed authors do (es) not meet the ICMJE criteria.

We believe these individuals should be listed as authors because:

[Please elaborate below]  

We confirm that the manuscript has been read and approved by all named authors.

We confirm that the order of authors listed in the manuscript has been approved by all named authors.

### Contact with the editorial office

The Corresponding Author declared on the title page of the manuscript is:

Careen Y. Lowder, M.D., PhD.

This author submitted this manuscript using his/her account in EVISE.

We understand that this Corresponding Author is the sole contact for the Editorial process (including EVISE and direct communications with the office). He/she is responsible for communicating with the other authors about progress, submissions of revisions and final approval of proofs.

We confirm that the email address shown below is accessible by the Corresponding Author, is the address to which Corresponding Author's EVISE account is linked, and has been configured to accept email from the editorial office of American Journal of Ophthalmology Case Reports:

[Insert email address you wish to use for communication with the journal here].

Someone other than the Corresponding Author declared above submitted this manuscript from his/her account in EVISE:

Rebecca I. Chen.

We understand that this author is the sole contact for the Editorial process (including EVISE and direct communications with the office). He/she is responsible for communicating with the other authors, including the Corresponding Author, about progress, submissions of revisions and final approval of proofs.

We the undersigned agree with all of the above. Author’s name (First, Last) Signature Date

1.Rebecca Chen Rebecca Chen 3/1/2020
2.Jordan Deaner Jordan Deaner 3/1/2020
3.Sunil Srivastava Sunil Srivastava 3/1/2020
4 Careen Lowder Careen Lowder 3/1/2020

## Authorship

All authors attest that they meet the current ICMJE criteria for Authorship.

## Declaration of competing interest

Potential conflict of interest exists

Bottom of Form.

We wish to draw the attention of the Editor to the following facts, which may be considered as potential conflicts of interest, and to significant financial contributions to this work:

•Consultant/Advisory Board: Bausch and Lomb, Allergan, Clearside, Regeneron, Santen, Sanofi, Zeiss, Optos.
•Research Grants: Bausch and Lomb, Allergan, Novartis, Clearside, Zeiss, Sanofi, Santen.
•Licensing Royalty: Bioptigen, Synergetics
•Grant Funding: Ohio Department of Development TECH-13-059

The following authors have no financial disclosures: RC, JD, CL.
